# Adaptation of leukemia cells to hypoxic condition through switching the energy metabolism or avoiding the oxidative stress

**DOI:** 10.1186/1471-2407-14-76

**Published:** 2014-02-10

**Authors:** Mineaki Goto, Hiroshi Miwa, Kazuto Suganuma, Norikazu Tsunekawa-Imai, Masato Shikami, Motonori Mizutani, Shohei Mizuno, Ichiro Hanamura, Masakazu Nitta

**Affiliations:** 1 Department of Internal Medicine, Division of Hematology, Aichi Medical University School of Medicine, 1-1 Yazakokarimata, Nagakute, Aichi 480-1195, Japan; 2 Department of Internal Medicine, Division of Hematology, Daiyukai General Hospital, Ichinomiya, Aichi 491-0113, Japan

**Keywords:** Leukemia, Hypoxia, Energy metabolism, Reactive oxygen species, Pyruvate dehydrogenase kinase, Cytochrome c oxidase

## Abstract

**Background:**

Like normal hematopoietic stem cells, leukemia cells proliferate in bone marrow, where oxygen supply is limited. However, the growth and energy metabolism of leukemia cells under hypoxia have not been well understood. Although it has been known that reactive oxygen species (ROS) is generated under hypoxic conditions, normal and leukemia stem cells were characterized by relatively low levels of ROS. Roles of ROS on leukemia cells under hypoxia also have not been well understood.

**Methods:**

Four Leukemia cell lines were cultured under normoxia (21% O_2_) or hypoxia (1% O_2_), where NB4 and THP-1 were most extensively studied. To evaluate energy metabolism, we estimated whole cell number or apoptotic cells with or without a glycolysis inhibitor or an oxidative phosphorylation (OXPHOS) inhibitor. Glucose consumption and lactate production were also measured. To evaluate oxidative stress in hypoxic condition, the ROS level and GSH (reduced glutathione) / GSSG (oxidized glutathione) ratio was measured. In addition, pyruvate dehydrogenase kinase 1 (PDK1) and cytochrome c oxidase subunit 4 (COX4) were examined by western blotting or RT-PCR.

**Results:**

NB4, which grows well under normoxia depending on glycolysis, demonstrated prominent apoptosis and growth suppression after 48 hours culture under hypoxia. NB4 cells cultured under hypoxia showed significantly increased ROS. Culture with a ROS scavenger resulted in decrease of apoptotic cell death of NB4 under hypoxia. NB4 cells cultured for longer period (7 days) under hypoxia did not come to extinction, but grew slowly by upregulating GSH synthesis to protect from ROS generated in hypoxic condition. By contrast, THP-1, which largely depends on OXPHOS in mitochondria under normoxia, demonstrated more growth under hypoxia by changing metabolism from OXPHOS to glycolysis through upregulating PDK1. Moreover, THP-1 avoided ROS generation by substituting COX 4 subunit (from COX 4–1 to COX 4–2) through upregulation of LON, a mitochondrial protease under hypoxia.

**Conclusions:**

We showed that leukemia cells survive and adapt to the hypoxic condition through various pathways. Our results will help understanding energy metabolism of leukemia cells and creating novel therapeutics.

## Background

Hematopoietic stem cells are localized in bone marrow, where oxygen supply is limited. Hematopoietic stem cells (HSCs) in hypoxic bone marrow have been demonstrated to generate ATPs by anaerobic glycolysis rather than mitochondrial oxidative phosphorylation [1,2]. It has recently been shown that characteristics of HSCs such as cell cycle quiescence and transplantation capacity are secured by glycolytic metabolic status through pyruvate dehydrogenase kinase (PDK)-dependent mechanism [3]. It has been demonstrated that the bone marrow microenvironment also plays a pivotal role in the initiation and propagation of leukemia. Leukemic cells can infiltrate the microenvironment (niche) and may hijack the homeostatic mechanisms of normal hematopoiesis, leading to enhanced self-renewal and proliferation, quiescence, and resistance to chemotherapeutic agents [4-6].

It has been demonstrated that reactive oxygen species (ROS) is generated by mitochondria under hypoxic conditions [7-10]. Like normal hematopoietic stem cells, leukemia stem cells are characterized by relatively low levels of ROS (ROS-low) [11-13]. However, ROS can modulate the activation of signal transduction pathways involved in cellular proliferation and differentiation [14]. We also examined the contribution of ROS to the growth of leukemia cells under hypoxia.

Recent studies have indicated that some cancer cells do not dependent on glycolysis but on oxidative phosphorylation (OXPHOS) in mitochondria [12,15]. Some reports indicated that cancer cell subsets with different dependencies in energy generating pathways coexist within tumors in a symbiotic manner [16,17].

We previously described that energy metabolism of leukemia cells under normoxia and found that some leukemia cell lines depended on glycolysis and others on OXPHOS [18-20]. In these reports, we noted the necessity of studying the energy metabolism under hypoxia such as bone marrow environment where leukemia cells proliferate, which has rarely been reported. Here, we examined the growth and energy metabolism of leukemia cells under hypoxia to clarify how leukemic cells survive and proliferate in hypoxic bone marrow.

## Methods

### Cell line

Four acute myelogenous leukemia (AML) cell lines were used in this study. NB4, a t(15;17) APL cell line, was provided by Dr. M. Lanotte (Saint Louis Hospital, France). Kasumi-1, a t(8;21) AML cell line, was provided by Dr. N. Kamada (Hiroshima University, Japan). THP-1, a monocytic AML cell line, and HL-60, a differentiative AML cell line were provided from Cell Resource Center for Biomedical Research (Tohoku University, Japan). It was confirmed that all cell lines were the same as the cells registered in Japanese Collection of Research Bioresources (JCRB) Cell Bank (Osaka, Japan), by the comparison with the database of JCRB cell bank. Cell lines were usually grown in RPMI1640 medium containing 10% fetal calf serum (FCS, Thermo Electron, Melbourne, Australia) in humidified atmosphere of 5% CO_2_ and 95% air at 37°C.

### Cell cultures

Cell line cells in logarithmic growth were cultured at the density of 2 × 10^5^/ml (0.5 ml RPMI1640 containing 3% FCS/well) in tissue culture plate, 24 wells (Becton Dickinson, NJ, USA) under normoxia (21% O_2_) or hypoxia (1% O_2_). Then, cell number was estimated at 24 hours and at 48 hours by Particle Analyzer (PA-2000, Erma Inc., Japan). Cell line cells were cultured in the same condition with various concentrations of 2-fluoro-2-deoxy-D-glucose (2-FDG: Sigma #F5006) (2, 5 and 10 mM) or oligomycin (oligo: Sigma #O4876) (0.05, 0.2 and 1 μg/ml) or N-acetyl-L-cysteine (NAC: Sigma #A7250) (0.5 mM). Control culture containing same amount of solvent (H_2_O for 2-FDG, DMSO for oligo) was done simultaneously. After 24 or 48 hours culture, cell number was estimated by Particle Analyzer.

### Enzymatic measurement of glucose, lactic acid

Cell lines were cultured in the same condition as in the section of Cell Cultures for 48 hours. Then, concentration of glucose and lactic acid in the culture supernatant was measured by using D-Glucose kit or L-Lactic acid kit (UV method: R-Biopharm AG, Darmstadt, Germany). Glucose consumption was calculated as follows: [glucose consumption (g/l)] = [glucose concentration of cell free medium = 1.92 (g/l)] – [measured glucose concentration after culture (g/l)]. Lactate production (g/l) was defined as measured lactate concentration after culture (g/l), since cell free medium did not contain lactate.

### Apoptosis assay

After 12 or 24 hours culture in the same condition as in the section of Cell Cultures, apoptosis of NB4 and THP-1 was examined by annexin V / propidium iodide (PI) staining (TACS Annexin V-FITC Apoptosis Detection Kit, TREVIGEN, Gaithersburg, MD), and analyzed by flow cytometer (FACS Canto II, BD Biosciences, San Jose, CA). Annexin V positive cells were defined as apoptotic cells, and annexin V negative and PI negative (annexin-/PI-) cells were defined as viable cells. The viable cell number with or without NAC (0.5 mM) were examined after 48 hours culture.

### ROS and GSH (reduced glutathione) / GSSG (oxidized glutathione) measurements

After 48 hours culture in the same condition as in the section of Cell Cultures with or without NAC (0.5 mM), CellROX Deep Red Reagent (Life Technologies Corporation, CA) (1 μM) was added and incubated for 30 min, then washed and analyzed by flow cytometry to detect ROS level.

After 24 hours culture at the density of 2 × 10^5^/ml (0.02 ml RPMI1640 containing 3% FCS/each well) in tissue cluture plate, 96 wells, U-bottom (Becton Dickinson, NJ, USA) under normoxia or hypoxia (short term hypoxia: SH), cells were collected and assayed for GSH / GSSG-Glo Assay (Promega, USA). After 7 days culture under hypoxia with two medium changes, GSH / GSSG measurement was also done using these cells cultured for 24 more hours under hypoxia (long term hypoxia: LH).

### Reverse transcriptase- polymerase chain reaction (PCR)

After 24 hours culture under normoxia or hypoxia, total RNA was isolated from NB4 and THP-1 using RNeasy Mini Kit (Qiagen, Germany). 1 μg of total RNA was reverse transcribed using random hexamer priming and SuperScriptIII reverse transcriptase (Life Technologies, Carlsbad, CA) to generate cDNA. Then, PCR was done using KOD FX Neo (TOYOBO, Osaka, Japan) with 50 ng cDNA in the following conditions: After predenature at 94°C for 2 min, 15 sec at 98°C, 30 sec at 53°C, 15 sec at 68°C (28 cycles) for COX 4 isoform 1 (4–1); After predenature at 94°C for 2 min, 15 sec at 98°C, 30 sec at 57°C, 20 sec at 68°C (38 cycles) for COX4 isoform 2 (4–2); After predenature at 94°C for 2 min, 15 sec at 98°C, 30 sec at 56°C, 20 sec at 68°C (28 cycles) for LON; After predenature at 94°C for 2 min, 10 sec at 98°C, 30 sec at 58°C, 18 sec at 68°C (25 cycles) for β-actin. Primers for PCR were as follows: COX 4–1 Fw 5′-GAGCAATTTCCACCTCTG-3′, Rev 5′-CAGGAGGCCTTCTCCTTCTC-3′, COX 4–2 Fw 5′-GCTATGCCCAGCGCTACTAC-3′, Rev 5′-CATCTCCGCAAAGGTCTCAT-3′, LON Fw 5′-CGGGAAGATCATCCAGTGTT-3′, Rev 5′-ACGTCCAGGTAGTGGTCCAG-3′ and β-actin (as a control gene) Fw 5′-CGGCGACGACCCATTCGAAC-3′, Rev 5′-GAATCGAACCCTGATTCCCCGTC-3′.

### Western blot

After 24 hours culture under normoxia or hypoxia, 1 × 10^6^ cells of NB4 or THP-1 were lysed in 0.1 ml Blue Loading Buffer Pack (Cell Signaling #7722, Danvers, MA) and 0.02 ml of lysed cells were electrophoresed in 7.5% polyacrylamide gel. The gel was electroblotted onto hybond-P (GE Healthcare, UK). The membrane was hybridized with antibody to PDK1 (mouse monoclonal antibody, IgG1: abcam #ab110335, UK) at 1:1000 dilution and reacted with anti-mouse IgG secondary antibody (Cell Signaling #7076, Danvers, MA) at 1:2000 dilution. The membrane was visualized by ECL-prime Detection System (GE Healthcare, UK). β-actin (horseradish peroxidase-conjugated rabbit monoclonal antibody, IgG: Cell Signaling #5125, Danvers, MA) was also tested as control.

### Statistical analysis

The statistical analyses were done by *t*-test (unpaired, two-tailed) using Graph Pad Prism software. Data were presented as mean ± SEM of three independent experiments.

## Results

### Proliferation of leukemia cell line cells under hypoxia versus normoxia

**Figure 1:**
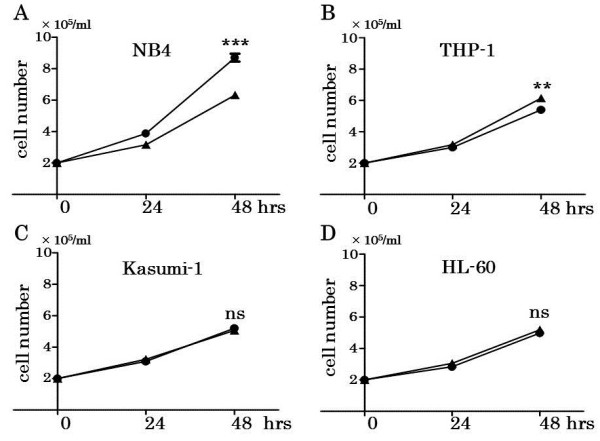
**Cell proliferation of four leukemia cell lines with no treatment under normoxia or hypoxia.** 2 × 10^5^/ml cells in 0.5 ml RPMI1640 + 3% FCS were cultured under normoxia (21% O_2_) or hypoxia (1% O_2_) for 48 hours in triplicate. Then, cell count was measured after 24 and 48 hours, respectively. NB4 **(A)** significantly diminished their proliferation under hypoxia compared with normoxia (48 hours) (p = 0.0001). In contrast, THP-1 **(B)** significantly increased more under hypoxia than normoxia (48 hours) (p = 0.005). Kasumi-1 **(C)** and HL-60 **(D)** did not show significant difference of cell growth between normoxia and hypoxia (48 hours). ▲: under hypoxia, ●: under normoxia. *** p < 0.001, ** p < 0.01, ns p > 0.05 (unpaired, two-tailed *t*-test).

To investigate the proliferation of leukemia cell line cells under hypoxia, the number of four leukemia cell line cells was estimated under normoxia or hypoxia after 24 and 48 hours incubation (Figure 1). NB4 grew more than other three leukemia cell lines under normoxia. However, the proliferation of NB4 under hypoxia was less than that under normoxia (p = 0.0001). After 48 hours culture, only THP-1 grew more under hypoxia than under normoxia (p = 0.005). HL-60 and Kasumi-1 did not show significant difference of growth after 48 hours culture under normoxia and hypoxia (HL-60 p = 0.1906; Kasumi-1 p = 0.1161). Then, we compared NB4 and THP-1 under hypoxia to understand the influence of hypoxia on leukemia cell growth.

### Apoptosis assay under hypoxia versus normoxia

**Figure 2:**
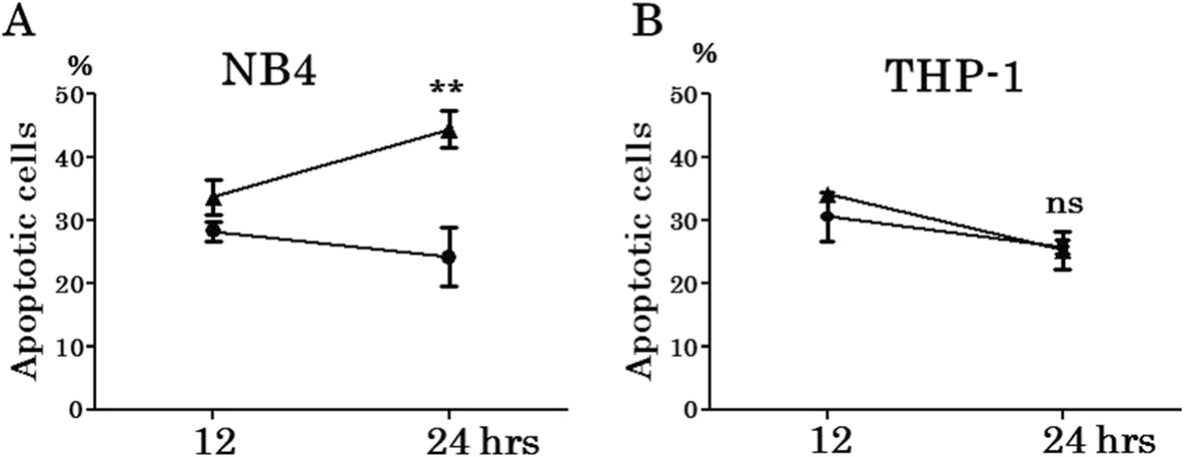
**Apoptosis assay by annexin V staining under hypoxia versus normoxia on NB4 and THP-1.** NB4 and THP-1 were cultured in the same condition as cell proliferation assay for 24 hours in triplicate. Then, apoptotic cells were examined after 12 and 24 hours by annexin V / PI method. Apoptotic cells of NB4 **(A)** under 24 hours hypoxia were significantly increased (p = 0.0031), although THP-1 **(B)** did not show increased apoptosis under hypoxia at least 24 hours (p = 0.7876). ▲: under hypoxia, ●: under normoxia. H: under hypoxia, N: under normoxia. *** p < 0.001, ns p > 0.05 (unpaired, two-tailed *t*-test).

After 24 hours culture under hypoxia, NB4 showed significant increase of apoptotic cells when compared with that under normoxia (p = 0.0031) (Figure 2A). However, there was no increase of annexin V positive cells in THP-1 after 24 hours culture under hypoxia (p = 0.7876) (Figure 2B).

### ROS measurement and the effects of ROS scavenger

**Figure 3:**
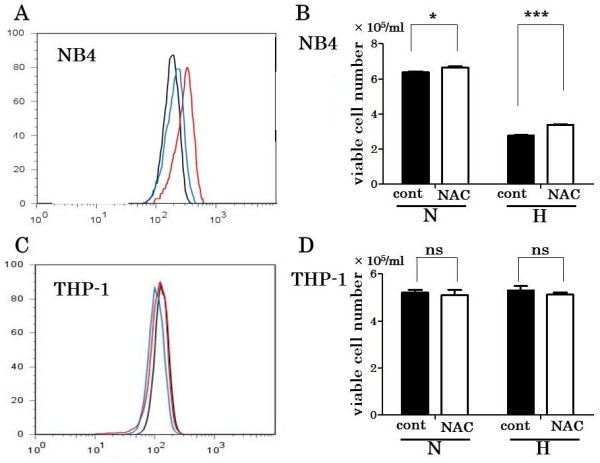
**ROS and its effects on the viability of NB4 and THP-1 under hypoxia.** NB4 and THP-1 were cultured in the same condition as cell proliferation assay for 48 hours with or without NAC 0.5 mM. ROS levels were measured by using CellROX Deep Red Reagent. The viable cell numbers of NB4 and THP-1 with or without NAC 0.5 mM were examined by annexin V / PI method in triplicate. ROS level of NB4 **(A)** was much higher under 48 hours hypoxia (red line) than normoxia (black line). Incubation with NAC made ROS level decreased in NB4 under hypoxia (blue line). NAC treatment increased viable cells cultured under hypoxia (p = 0.0002) (**B** right), although the effect of NAC was not so obvious under normoxia (p = 0.0285) (**B** left). On the other hand, THP-1 did not show ROS increase under hypoxia and addition of NAC did not work as same as NB4 **(C, D)**. black line: ROS level under normoxia as control, red line: under hypoxia, blue line: under hypoxia with NAC. H: under hypoxia, N: under normoxia. *** p < 0.001, * p < 0.05, ns p > 0.05 (unpaired, two-tailed *t*-test).

Since some reports indicated that ROS was increased under hypoxia, we determined the ROS level of NB4 and THP-1 under normoxia and hypoxia. ROS level was much higher in NB4 under 48 hours hypoxia than normoxia. Moreover, incubation with NAC as ROS scavenger made ROS level decreased in NB4 under hypoxia (Figure 3A). In addition, NAC treatment increased viable cells cultured under hypoxia (p = 0.0002), although the effect of NAC was not so obvious under normoxia (p = 0.0285) (Figure 3B). On the other hand, THP-1 did not show ROS increase under hypoxia and addition of NAC did not work as same as NB4 (Figure 3C and D).

### GSH / GSSG measurement

**Figure 4:**
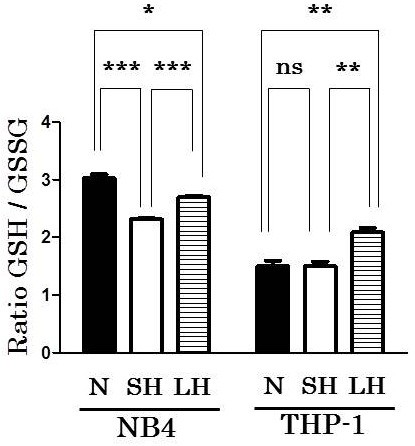
**GSH / GSSG ratio measurement under normoxia and two different terms of hypoxia.** 2 × 10^5^/ml cells in 0.02 ml RPMI1640 + 3% FCS were cultured under normoxia (21% O_2_) or hypoxia (1% O_2_) (short term hypoxia) for 24 hours in triplicate. Then, GSH / GSSG ratio was measured by using GSH / GSSG-Glo Assay. After 7 days culture under hypoxia with two medium changes, GSH / GSSG ratio was also measured by using these cells cultured for 24 more hours under hypoxia (long term hypoxia). GSH / GSSG ratio of NB4 under normoxia was 3.02 ± 0.067 and that under short term hypoxia was 2.31 ± 0.020 (p = 0.0005). GSH / GSSG ratio under long term hypoxia (2.69 ± 0.028) was significantly higher than that of short term hypoxia (p = 0.0004). GSH / GSSG ratio of THP-1 under normoxia (1.50 ± 0.094) and hypoxia (1.50 ± 0.083) was almost equal. THP-1 under longer hypoxia showed increase of GSH / GSSG ratio (2.09 ± 0.077) compared with that under short term hypoxia (p = 0.0065). SH: under short term hypoxia, LH under long term hypoxia, N: under normoxia. *** p < 0.001, ** p < 0.01, * p < 0.05, ns p > 0.05 (unpaired, two-tailed *t*-test).

To evaluate oxidative stress such as ROS, we performed GSH / GSSG ratio measurement. We compared normoxia to hypoxia (short term or long term) in NB4 and THP-1. GSH / GSSG ratio of NB4 under normoxia was 3.02 ± 0.067 and that of NB4 under hypoxia was 2.31 ± 0.020, suggesting that NB4 under hypoxia had much oxidative stress. GSH / GSSG ratio of NB4 under long term (7 days) hypoxia (the ratio was 2.69 ± 0.028) was higher than short term (24 hours) hypoxia (the ratio was 2.31 ± 0.020). GSH / GSSG ratio of THP-1 under normoxia (1.50 ± 0.094) and hypoxia (1.50 ± 0.083) was almost equal, suggesting that THP-1 had equal oxidative stress regardless of oxygen concentration. THP-1 under longer hypoxia showed increase of GSH / GSSG ratio (the ratio was 2.09 ± 0.077) (Figure 4).

### Glucose consumption and lactate production

**Figure 5:**
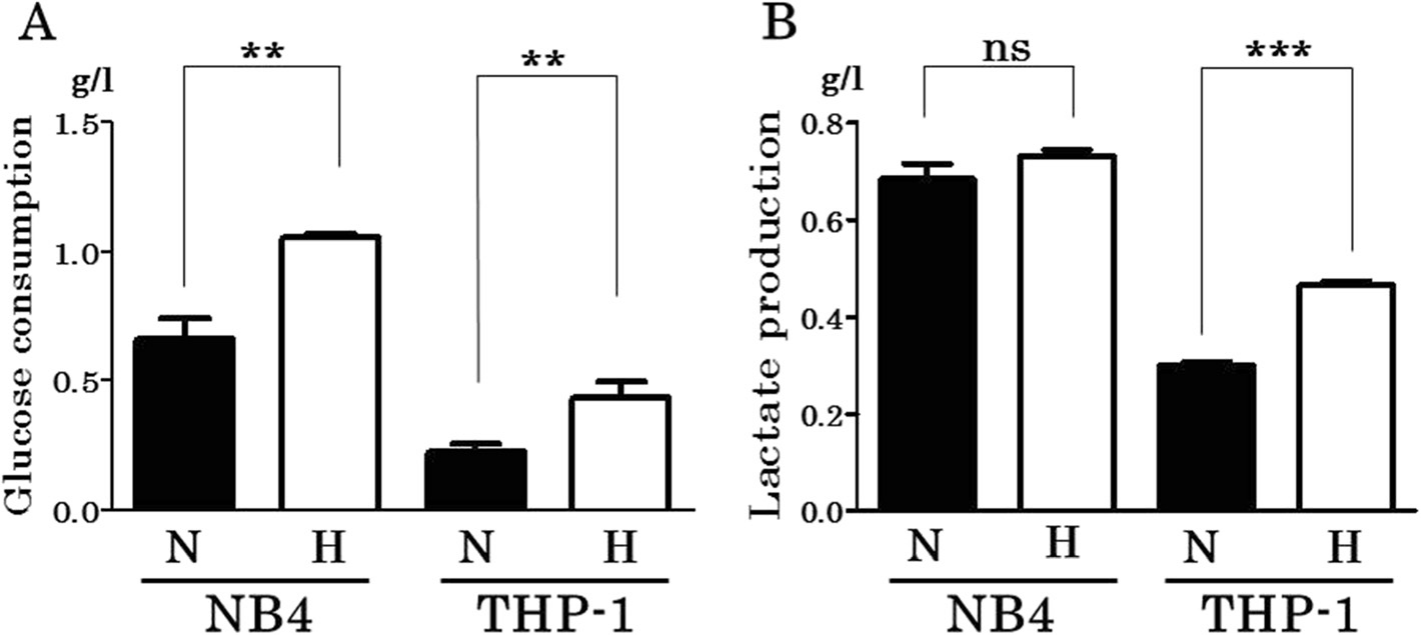
**Glucose consumption and lactate production in NB4 and THP-1 under normoxia versus hypoxia.** NB4 and THP-1 were cultured in the same condition as cell proliferation assay for 48 hours in triplicate. Then, concentration of glucose and lactic acid in the culture supernatant was measured by using D-Glucose kit or L-Lactic acid kit. Both cell lines showed increased glucose consumption under hypoxia compared with normoxia with statistical significance (p = 0.0013 in NB4, p = 0.0054 in THP-1) **(A)**. THP-1 demonstrated increased lactate production under hypoxia (p < 0.0001), but NB4 did not (p = 0.0663) **(B)**. H: under hypoxia, N: under normoxia. *** p < 0.001, ** p < 0.01, ns p > 0.05 (unpaired, two-tailed *t*-test).

Glucose consumption of NB4 under normoxia (for 48 hours) was 0.6589 ± 0.04879 g/l and that under hypoxia was 1.056 ± 0.00552 g/l, respectively. In THP-1, glucose consumption under normoxia (for 48 hours) was 0.2258 ± 0.01804 g/l and that under hypoxia was 0.4366 ±0.03404 g/l, respectively. Both cell lines had significant difference between normoxia and hypoxia in glucose consumption (p = 0.0013 in NB4, p = 0.0054 in THP-1) (Figure 5A). Then, lactate production of NB4 under normoxia (for 48 hours) was 0.6847 ± 0.01780 g/l and that under hypoxia was 0.7323 ± 0.006692 g/l, which were not significantly different (p = 0.0663). In THP-1, lactate production under normoxia (for 48 hours) was 0.3010 ± 0.003512 g/l and that under hypoxia was 0.4663 ± 0.004096 g/l, where the difference was significant (p < 0.0001) (Figure 5B).

### Growth inhibition with 2-FDG or oligo under hypoxia versus normoxia

**Figure 6:**
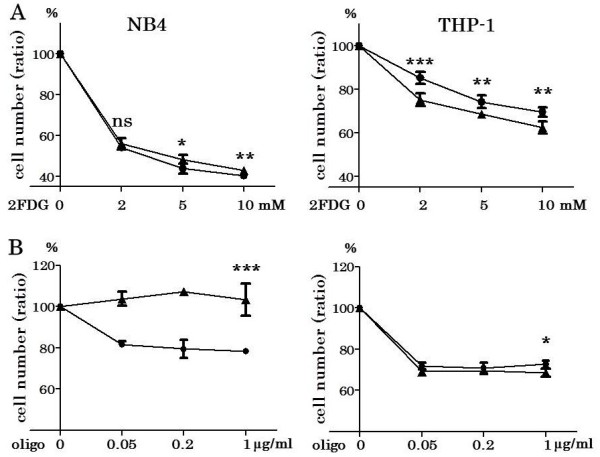
**Growth inhibition by 2-FDG or oligo on NB4 and THP-1 under normoxia and hypoxia.** NB4 and THP-1 were cultured in the same condition as cell proliferation assay for 48 hours with 2-FDG (0, 2, 5 10 mM) or oligo (0, 0.05, 0.2, 1 μg/ml) in triplicate. The cell numbers after 48 hours culture under normoxia or hypoxia with 2-FDG or oligo were divided by cell numbers of control (average of triplicated experiments) to obtain cell number ratios, in which the cell number with no treatment corresponded to 100%. The growth suppression of THP-1 by 2-FDG even at 2 mM under hypoxia was significantly more pronounced than that under normoxia (p = 0.0005), although the growth inhibition of NB4 by 2-FDG under hypoxia was slightly less than that under normoxia **(A)**. Oligo did not suppress the growth of NB4 under hypoxia, but suppressed that under normoxia (at 1 μg/ml, p < 0.0001). Oligo similarly suppressed the growth of THP-1 under normoxia and hypoxia **(B)**. ▲: under hypoxia, ●: under normoxia. *** p < 0.001, ** p < 0.01, ns p > 0.05 (unpaired, two-tailed *t*-test).

The cell count assay with 2-FDG as a glycolysis inhibitor was performed on NB4 and THP-1 under normoxia and hypoxia. The growth suppression of THP-1 by 2-FDG even at 2 mM under hypoxia was significantly more pronounced than that under normoxia, although the growth inhibition of NB4 by 2-FDG under hypoxia was slightly less than that under normoxia (Figure 6A).

The cell count assay with oligo as a mitochondrial oxidative phosphorylation inhibitor was performed on NB4 and THP-1 under normoxia and hypoxia. Oligo did not suppress the growth of NB4 under hypoxia, but suppressed that under normoxia (p < 0.0001). Oligo similarly suppressed the growth of THP-1 under normoxia and hypoxia (Figure 6B).

### Western blotting for detecting upregulation of PDK1

**Figure 7:**
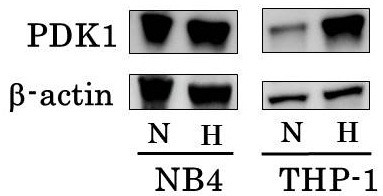
**Western blotting of PDK1.** NB4 and THP-1 were cultured in the same condition as cell proliferation assay for 24 hours. PDK1 was abundantly expressed in NB4 even under normoxia, culture under hypoxia did not augment the expression of PDK1 in NB4. Expression of PDK1 was augmented by hypoxia in THP-1. Top, PDK1; bottom, β-actin. H: under hypoxia, N: under normoxia.

PDK1 inactivates pyruvate dehydrogenase (PDH) complex and leads to suppression of the influx of glycolytic metabolites into mitochondria. PDK1 is induced by hypoxia in normal cell for adaptation to hypoxia and often expressed constantly in some cancer. Western blot showed that PDK1 was abundantly expressed in NB4 even under normoxia, and culture under hypoxia did not augment the expression of PDK1 in NB4. Expression of PDK1 was augmented by hypoxia in THP-1, which was demonstrated by western blotting (Figure 7).

### mRNA expression of COX 4 and mitochondrial protease LON

**Figure 8:**
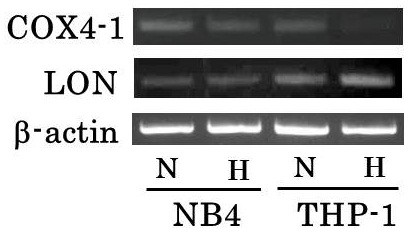
**RT-PCR analysis of COX 4–1 and LON on NB4 and THP-1 under normoxia or hypoxia.** NB4 and THP-1 were cultured in the same condition as cell proliferation assay for 24 hours. The expression of COX 4–1 in THP-1 under hypoxia was decreased compared with normoxia. In addition, mRNA expression of LON in THP-1 under hypoxia was increased compared with that in normoxia. In NB4, mRNA expression of COX 4–1 and LON was not influenced by O_2_ concentration. β-actin was performed as an internal control. Top, COX 4–1; middle, LON; bottom, β-actin. H: under hypoxia, N: under normoxia.

It has been demonstrated that expression of the COX 4–1 and 4–2 isoforms is O_2_ regulated. Under hypoxia, isoform is switched from COX 4–1 to COX 4–2 by inducing LON, a mitochondrial protease that is required for COX 4–1 degradation to optimize the efficiency of respiration at low O_2_ concentration [21]. As shown in Figure 8, mRNA expression of COX 4–1 in THP-1 under hypoxia was decreased compared with that in normoxia. In addition, mRNA expression of LON in THP-1 under hypoxia was increased compared with that in normoxia. In this experiment, mRNA expression of COX 4–2 could not be detected even repeated experiments. In NB4, mRNA expression of COX 4–1 and LON was not influenced by O_2_ concentration.

## Discussion

Recently, inhibiting glycolysis draws attention as novel cancer therapeutics. Since there are alternative pathways to overcome energy depletion by glycolysis inhibition [12,15], the effectiveness of anti-glycolysis therapy alone is controversial. We thought therapeutic strategies targeting leukemia cell metabolism would be improved by studying energy metabolism more precisely, especially under hypoxia where leukemia cells grow.

We examined the proliferation of leukemia cell line cells under hypoxia and compared with that under normoxia. NB4, an APL cell line, which mainly depends on glycolysis for energy production [18-20], demonstrated prominent apoptosis and growth suppression after 48 hours culture under hypoxia. It was contrary to our expectation that NB4, already dependent on glycolysis, would proliferate well under hypoxia. However, NB4 utilizes OXPHOS in mitochondria to some extent for energy production, which is validated by growth suppression with oligo treatment. Then, we examined the ROS, since it has been demonstrated that ROS is generated by mitochondria under hypoxic conditions [7-10] and checked if apoptosis induced by hypoxia is caused by increased ROS. NB4 cells cultured under hypoxia showed significantly increased ROS and culture with NAC, a ROS scavenger or oligo, an inhibitor of OXPHOS in mitochondria resulted in decrease of apoptotic cell death of NB4 under hypoxia. However, leukemia cells proliferate in hypoxic bone marrow. NB4 cells cultured for longer period (7 days) under hypoxia did not come to extinction, but grew slowly. NB4 cells cultured under hypoxia for longer period showed improved GSH / GSSG ratio compared with shorter hypoxia. Collectively, NB4 can survive and proliferate in hypoxic bone marrow by upregulating GSH synthesis to protect from ROS generated in hypoxic condition.

THP-1 showed more growth under hypoxia than normoxia. THP-1 largely depended on OXPHOS in mitochondria when grown under normoxia [18-20]. However, we demonstrated that THP-1 changed the main energy metabolism from OXPHOS to glycolysis by upregulating PDK1 under hypoxia. THP-1 consumed more glucose and produced more lactate under hypoxia than normoxia, and the growth of THP-1 was significantly more suppressed by a glycolysis inhibitor, 2-FDG under hypoxia. However, oligo suppressed the growth of THP-1 under hypoxia to the almost same extent as under normoxia, which means that OXPHOS in mitochondria is still functional under hypoxia. If OXPHOS in mitochondria is functional under hypoxia, ROS would be produced to induce apoptosis like NB4. However, ROS was not increased under hypoxia in THP-1. We thought that THP-1 has mechanisms to avoid ROS generation under hypoxia. Since Fukuda et al. demonstrated that cancer cells survive hypoxic condition by COX 4 subunit exchange to minimize ROS production, we examined COX 4 system. We found that in THP-1 under hypoxia, expression of COX 4–1 was decreased and LON, a mitochondrial protease that might be required for COX 4–1 degradation, is upregulated (switched from COX 4–1 to COX 4–2) to avoid ROS generation by efficient electron transport under hypoxia. We could not detect COX 4–2 expression, which might be due to paucity of expression in hematopoietic cells [22].

From our observation described here, leukemia cells survive environmental change such as hypoxia by various mechanisms. Determining detailed metabolic system would lead to novel leukemia therapy suitable for each leukemia cells, for example, inhibition of GSH production for NB4 and inhibition of LON protease for THP-1 under hypoxia.

## Conclusions

We have demonstrated that leukemia cells do not always depend on glycolysis for energy production but on OXPHOS, and present study have shown that leukemia cells survive and adapt to the hypoxic condition through various pathways; scavenging ROS or metabolism switch by upregulating PDK1 and COX 4 subunit exchange. The concept of targeting cancer metabolism has emerged as an intriguing approach to the development of improved therapeutic regimens. Understanding further the metabolic regulation of leukemia cells may yield improved therapeutic strategies.

## Abbreviations

AML: Acute myelogenous leukemia; COX: Cytochrome c oxidase; FCS: Fetal calf serum; GSH: Reduced glutathione; GSSG: Oxidized glutathione; HSCs: Hematopoietic stem cells; NAC: N-acetyl-L-cysteine; oligo: Oligomycin; OXPHOS: Oxidative phosphorylation; PDH: Pyruvate dehydrogenase; PDK: Pyruvate dehydrogenase kinase; PI: Propidium iodide; ROS: Reactive oxygen species; RT-PCR: Reverse transcriptase-polymerase chain reaction; 2-FDG: 2-fluoro-2-deoxy-D-glucose.

## Competing interests

The authors declare that they have no competing financial interests.

## Authors’ contributions

MG, KS, NT-I and MS performed experiments. MG and HM designed the research, carried out statistical analyses and wrote the manuscript. MM, SM, IH and MN participated in its design and coordination and helped to draft the manuscript. All authors read and approved the final manuscript.

## Pre-publication history

The pre-publication history for this paper can be accessed here:

http://www.biomedcentral.com/1471-2407/14/76/prepub
